# Supplementary material for: CryoEM structure of Drosophila flight muscle thick filaments at 7 Å resolution
Source: Life Sci Alliance. 2020 Jul 27;3(8):e202000823. doi: 10.26508/lsa.202000823 (PMC7391215; doi:10.26508/lsa.202000823)
Supplement: Supplemental Data 3. — Supplemental methods. [file LSA-2020-00823_Supplemental_Data_3.docx]

Supplementary Materials for

CryoEM Structure of Drosophila Flight Muscle Thick Filaments at 7Å Resolution

Nadia Daneshparvar, Dianne W. Taylor, Thomas S. O’Leary, Hamidreza Rahmani, Fatemeh Abbasi Yeganeh, Michael J. Previs, and Kenneth A. Taylor*

*Corresponding author. Email: taylor@bio.fsu.edu (K.A.T.)

# Materials and Methods

**Key Resources Table**

| REAGENT or RESOURCE | SOURCE | IDENTIFIER |
| --- | --- | --- |
| Chemicals, peptides, and recombinant proteins | | |
| Calcium insensitive gelsolin | M. Briggs, Duke University |  |
| Talon Metal affinity resin | Clontech | #635503 |
| LB Media | Fluka | #L31562 |
| Carbenicillin | Sigma | #C1389 |
| Doxycycline | Sigma | #D9191 |
| IPTG | Sigma | #15502 |
| RapiGest SF Surfactant | Waters Corporation |  |
| Porcine Erythrocyte Calpain | Athens Research & Technology, Athens, GA | # 16-05-030112-P |
|  |  |  |
| EXPERIMENTAL MODELS: ORGANISMS/STRAINS | | |
| *D. melanogaster* | Prof. Wu-Min Deng, Florida State Univ. | W1118 |
| *D. melanogaster* | Prof. J. Vigoreaux, Univ. Vermont | Dmlc2[Δ2-46; S66A,S67A] |
| *E.coli*: BL21(DE3) Competent cells | Novagen |  |
|  |  |  |
| DEPOSITED DATA | | |
| *Lethocerus* thick filament | EMD-3301 | (Hu et al., 2016) |
| Titan Fibronectin3 repeat | PDB code 1TTG | (Main et al., 1992) |
| Titan Ig domain | PDB code 1TIT | (Improta et al., 1996) |
| Myosin Binding ProteinC | PDB code 2YXM | (Kishishita) |
|  |  |  |
| SOFTWARE AND ALGORITHMS | | |
| MotionCor2 | <https://emcore.ucsf.edu/ucsf-motioncor2> | (Zheng et al., 2017) |
| CTFFIND4 | <http://grigoriefflab.janelia.org/ctffind4> | (Rohou and Grigorieff, 2015) |
| GCTF | <https://www.mrc-lmb.cam.ac.uk/kzhang/> | (Zhang, 2016) |
| RELION | [https://www3.mrc-lmb.cam.ac.uk/relion//index.php/Main_Page](https://www3.mrc-lmb.cam.ac.uk/relion/index.php/Main_Page) | (He and Scheres, 2017) |
| cisTEM | <https://cistem.org/> | (Grant et al., 2018) |
| EMAN2 | <http://blake.bcm.tmc.edu/EMAN2/> | (Tang et al., 2007) |
| UCSF Chimera | <https://www.cgl.ucsf.edu/chimera/> | (Pettersen et al., 2004) |

**Contact for Reagent and Resource Sharing**

Further information and requests for resources and reagents should be directed to and will be fulfilled by the Lead Contact, Kenneth A. Taylor (taylor@bio.fsu.edu)

**ADDITIONAL METHOD DETAILS**

**Protein Expression and Purification**

Calcium insensitive human plasma gelsolin (residues 25-406) was cloned and overexpressed in *Escherichia coli* BL21 (DE3) strain in Lysogeny Broth (LB) medium. The expression vector was obtained from Dr. Margaret Briggs (Duke University Medical Center). The protocol is as follows:

**Buffers for Gelsolin preparation**

1. LYSIS BUFFER (1X): 50 mM Tris, 0.1M (NaCl, 2mM EDTA with 0.1mM PMSF, 1mM DTT and 0.2mg/ ml lysozyme added, pH 7.9.
2. BINDING BUFFER (1X): 20 mM Tris, 50 mM NaCl, 5 Imidazole with 0.1 mM PMSF, and 6M Urea, pH 7.9.
3. WASH BUFFER (1X): 20 mM Tris, 50mM NaCl, 60 Imidazole + 6M Urea, pH 7.9
4. ELUTION BUFFER (1X): 20mM Tris, 50mM NaCl, 1M Imidazole + 6M Urea, pH 7.9
5. DIALYSIS BUFFER (1X): 10mM Mops, 20mM KCl, 5mM MgCl2, 5mM EGTA, 0.5mM DTT, pH 6.8. Need 3 liters in total.

**Gelsolin Preparation**

Transformed cells were used to seed 80 ml of LB media + 0.1ml Carbinicillin and 50 μl Doxycycline. Culture was grown over night at 35° C and 242 rpm. The following day 30 ml of overnight culture is added to 500 ml LB + 0.5ml Carbinicillin and 1.0 ml Doxycycline and grown at 242 rpm and 35 °C for 3 hrs. The Optical Density (OD) is checked and gel samples made until an OD between 0.6 and 0.7 is reached at which point 400 μl of 1 M IPTG is added and gel samples at 1, 2, and 3 hours post IPTG.

At 3 hrs. the culture is centrifuged at 8K rpm, 4°C, for 15 min to pellet the cells. The supernatant is poured off and the pellet resuspended in PBS and transfered to a 50 ml centrifuge tube. After centrifuging at 7000 rpm, 4°C for 15 min, the pelleted cells are stored at -80°C.

For protein isolation, the pellet was thawed slowly on ice and suspended in 6 ml Lysis buffer +6 mg dry lysozyme. The thawed pellet was then shaken for 20 min at 250 rpm at 25°C.

Lysis buffer was then added to 25 ml and the suspension sonicated until large aggregates are no longer visible. The suspension, which consists primarily of gelsolin in inclusion bodies, was then transfer to 50 ml centrifuge tube and centrifuged at 11.5 rpm for 15 min. The pellet is resuspended in 20 ml Binding buffer plus 0.1 mM PMSF and 0.5% Triton and left for 20 min on ice. The suspension is pelleted by centrifugation at 20,300 x g for 15 min. Resuspended in binding buffer and repelleted. Finally, the pellet is resuspended in 5 ml Binding buffer + 6M Urea.

To purify the gelsolin, first charge a Talon column with about 70 ml 50 mM CoCl_2_. Run 100 ml Binding buffer + 6M Urea.

Clarify the denatured gelsolin by centrifuging in a TLA100.3 rotor at 50,000 rpm for 30 min and load the supernatant on Talon column. Run 100 ml Binding buffer +10 mM Imidazole +6M Urea first to elute impurities followed by Binding buffer + 4 M imidazole + 6 M Urea. Take gel samples at regular intervals to check if gelsolin is not binding. After about 11 ml of 4M imidazole collect 2 ml fractions.

Read OD of fractions and save those fractions with the highest OD. Dialyze those in 1 liter Dialysis buffer with 3 changes. Centrifuge at 60,000x g, 30 min to clarify. Flash freeze small aliquots and store at -80°C.

**Buffers for Thick Filament Preparation**

1. RELAXING BUFFER (1X): 20 mM Na_2_HPO_4_, 80 mM KCl, 5 mM MgAcetate, 5 mM ATP, 5 mM EGTA, 1 mM DTT, pH 6.8.
2. CALPAIN BUFFER (1X): 10 mM Mops, 10 mM Na_2_HPO_4_, 80 mM KCl, 5 mM MgAcetate, 5 mM ATP, 5 mM EGTA, 3 mM DTT, 5.2 mM CaCl_2_, pH 6.8.
3. SHEAR BUFFER (1X): 20 mM MOPS, 20 mM Na_2_HPO_4_, 100 mM NaCl, 5 mM MgAcetate, 5 mM ATP, 5 mM EGTA. 5 mM DTT, pH 6.8
4. STOP BUFFER (1X): 20 mM MOPS, 150 mM NaCl, 5 mM MgAcetate, 15 mM EGTA, pH 6.8.

Flight muscle from the thoraces of ~10 files is deposited in 0.3 ml of relaxing buffer +10 μl protease inhibitor (Sigma 2714). Muscle is then homogenized in a 1 ml ground glass homogenizer to a total volume of 1 ml (with rinses) and transfered to 1.5 ml centrifuge tube. Myofibrils are separated from solubilized proteins by centrifugation at 6,000x g with turns of the tube to promote pelleting. (3-4 turns, 3 min each). The pellet is resuspended in 0.3 ml of relaxing buffer +0.5% Triton and incubated on ice for 15-30 min. After incubation in relaxing buffer +Triton, the myofibril suspension is centrifuged at 6,000x g with tube turns (3-4 turns, 3 min each), resuspended in relaxing buffer and centrifuged again without the incubation time.

The pellet is then resuspended in 0.1 ml Calpain buffer +1 μl calpain (Athens Research). Digest at r.t. for 1 hour. Digest is stopped by adding 0.2 ml Stop buffer.

Digested myofibrils are then separated by centrifuging at 7,000x g, with turns (4 turns, 3 min each), the supernatant discarded, and 35-80 μl of Shear buffer depending on pellet size. Myofibrils are sheared 10X by pulling prep through a 1 ml syringe with 26G needle. Large solids are removed by centrifuging at 3,500x g with 2 turns (2 turns, 2 min each) to remove solids. The supernatant is collected and mixed with gelsolin. Typically 15 μl gelsolin (concentration 2 mg/ml) is used.

Thick filaments were checked quality and concentration by negative staining using 2% uranyl acetate. Large undigested material was removed by low speed centrifugation. Thick filaments are never subjected to high-speed centrifugation to separate them from intact or partially digested actin filament fragments or to concentrate them for EM grid preparation. If the thick filament suspension is too dilute, successive drops are deposited on the EM grid.

**Processed Mass Spectrometry Data**

Two files of processed mass spectrometry data are included. The initial visualization in Excel shows only the individual proteins. The hidden intervening rows, if shown, contain the results for the individual trypsin peptides.

The raw data can be found at <ftp://massive.ucsd.edu/MSV000085627/>.

**References Cited**

Farman, G.P., M.S. Miller, M.C. Reedy, F.N. Soto-Adames, J.O. Vigoreaux, D.W. Maughan, and T.C. Irving. 2009. Phosphorylation and the N-terminal extension of the regulatory light chain help orient and align the myosin heads in Drosophila flight muscle. *J Struct Biol*. 168:240-249. 10.1016/j.jsb.2009.07.020

Gomez-Blanco, J., J.M. de la Rosa-Trevin, R. Marabini, L. Del Cano, A. Jimenez, M. Martinez, R. Melero, T. Majtner, D. Maluenda, J. Mota, et al. 2018. Using Scipion for stream image processing at Cryo-EM facilities. *J Struct Biol*. 204:457-463. 10.1016/j.jsb.2018.10.001

Grant, T., A. Rohou, and N. Grigorieff. 2018. cisTEM, user-friendly software for single-particle image processing. *Elife*. 7. 10.7554/eLife.35383

He, S., and S.H.W. Scheres. 2017. Helical reconstruction in RELION. *J Struct Biol*. 198:163-176. 10.1016/j.jsb.2017.02.003

Hu, Z., D.W. Taylor, M.K. Reedy, R.J. Edwards, and K.A. Taylor. 2016. Structure of myosin filaments from relaxed Lethocerus flight muscle by cryo-EM at 6 Å resolution. *Sci Adv*. 2:e1600058. 10.1126/sciadv.1600058

Improta, S., A.S. Politou, and A. Pastore. 1996. Immunoglobulin-like modules from titin I-band: extensible components of muscle elasticity. *Structure*. 4:323-337. 10.1016/s0969-2126(96)00036-6

Kishishita, S., Ohsawa, N., Murayama, K., Terada, T., Chen, L., Liu, Z., Shirouzu, M., Wang, B., Yokoyama, S. Crystal structure of I-set domain of human Myosin Binding ProteinC. <http://www.rcsb.org/structure/2YXM>.

Main, A.L., T.S. Harvey, M. Baron, J. Boyd, and I.D. Campbell. 1992. The three-dimensional structure of the tenth type III module of fibronectin: an insight into RGD-mediated interactions. *Cell*. 71:671-678. 10.1016/0092-8674(92)90600-h

Pettersen, E.F., T.D. Goddard, C.C. Huang, G.S. Couch, D.M. Greenblatt, E.C. Meng, and T.E. Ferrin. 2004. UCSF Chimera--a visualization system for exploratory research and analysis. *J Comput Chem*. 25:1605-1612. 10.1002/jcc.20084

Reedy, M.C., B. Bullard, and J.O. Vigoreaux. 2000. Flightin is essential for thick filament assembly and sarcomere stability in Drosophila flight muscles. *J. Cell Biol.* 151:1483-1500.

Rohou, A., and N. Grigorieff. 2015. CTFFIND4: Fast and accurate defocus estimation from electron micrographs. *J Struct Biol*. 192:216-221. 10.1016/j.jsb.2015.08.008

Tang, G., L. Peng, P.R. Baldwin, D.S. Mann, W. Jiang, I. Rees, and S.J. Ludtke. 2007. EMAN2: an extensible image processing suite for electron microscopy. *J Struct Biol*. 157:38-46. 10.1016/j.jsb.2006.05.009

Zhang, K. 2016. Gctf: Real-time CTF determination and correction. *J Struct Biol*. 193:1-12. 10.1016/j.jsb.2015.11.003

Zheng, S.Q., E. Palovcak, J.P. Armache, K.A. Verba, Y. Cheng, and D.A. Agard. 2017. MotionCor2: anisotropic correction of beam-induced motion for improved cryo-electron microscopy. *Nat Methods*. 14:331-332. 10.1038/nmeth.4193
